# Study on Spatiotemporal Evolution Features and Affecting Factors of Collaborative Governance of Pollution Reduction and Carbon Abatement in Urban Agglomerations of the Yellow River Basin

**DOI:** 10.3390/ijerph20053994

**Published:** 2023-02-23

**Authors:** Zhaoxian Su, Yang Yang, Yun Wang, Pan Zhang, Xin Luo

**Affiliations:** 1 School of Public Administration, North China University of Water Resources and Electric Power, Zhengzhou 450046, China; 2 School of Management and Economics, North China University of Water Resources and Electric Power, Zhengzhou 450046, China; 3 Institute of Geophysics, China Earthquake Administration, Beijing 100081, China

**Keywords:** pollution reduction and carbon abatement, urban agglomerations, composite system synergy model, the Yellow River Basin

## Abstract

Exploring spatiotemporal evolution features and factors affecting pollution reduction and carbon abatement on the urban agglomeration scale is helpful to better understand the interaction between ecological environment and economic development in urban agglomerations. In this study, we constructed an evaluation index system for collaborative governance of pollution reduction and carbon abatement in urban agglomerations. In addition, we employed the correlation coefficient matrix, the composite system synergy model, the Gini coefficient, and the Theil index to evaluate the level of and regional differences in collaborative governance of pollution reduction and carbon abatement in seven urban agglomerations in the Yellow River Basin from 2006 to 2020. Moreover, we explored the factors affecting collaborative governance of pollution reduction and carbon abatement in urban agglomerations in the basin. The following findings were obtained: (1) the order degree of collaborative governance of pollution reduction and carbon abatement in the seven urban agglomerations exhibited a significant growing trend, representing a spatial evolution feature of “high in the west and low in the east”; (2) the internal differences in collaborative governance synergy of pollution reduction and carbon abatement decreased in Lanzhou–Xining Urban Agglomeration, Hohhot–Baotou–Ordos–Yulin Urban Agglomeration, Central Shanxi Urban Agglomeration, Zhongyuan Urban Agglomeration, and Shandong Peninsula Urban Agglomeration, while the internal differences basically remained stable in Guanzhong Urban Agglomeration and the Urban Agglomeration along the Yellow River in Ningxia; (3) the variances in environmental regulation and industrial structure among urban agglomerations had a significant positive effect on collaborative governance of pollution reduction and carbon abatement in urban agglomerations in the basin, and the variances in economic growth had a significant inhibitory effect. In addition, the variances in energy consumption, greening construction, and opening-up had an inhibitory impact on collaborative governance of pollution reduction, but the impact was not significant. Finally, this study proposes various recommendations to improve collaborative governance for pollution reduction and carbon abatement in urban agglomerations in the basin in terms of promoting industrial structure upgrading, strengthening regional cooperation, and reducing regional differences. This paper represents an empirical reference for formulating differentiated collaborative governance strategies for pollution reduction and carbon abatement, comprehensive green and low-carbon economic and social transformation programs, and high-quality green development paths in urban agglomerations, which is of certain theoretical and practical significance.

## 1. Introduction

As the world economy rebounds from the COVID-19 crisis, the current trend of relying on coal to promote economic growth is continuing, and global energy related to CO_2_ emissions is exhibiting an upward trajectory. The sixth IPCC report issues the most severe warning so far: human behavior is accelerating the process of global warming at an alarming rate, and the global climate, oceans, and atmosphere are facing imminent and terrible risks [1]. In accordance with data recently published by IEA, China is currently one of the largest CO_2_ emitters in the world and predominant in changing the global climate [2]. Moreover, based on the EPI Assessment in 2022, China’s air quality ranked 160th among 180 countries and regions, and poor air quality poses a serious threat to public life and health [3]. The relevant research reveals that haze pollution has a negative environmental impact on residents’ health [4], urban crime rates [5], personal well-being [6], regional innovation vitality [7], and so forth.

Both CO_2_ and air pollutants mainly originate from combustion of fossil fuels in human production and life. Because of their homology and synchronization in terms of the production process and the mutual effect of the hazard generation process, collaborative governance of pollution reduction and carbon abatement (PRCA) is theoretically feasible [8,9]. Based on the previous literature, it was found that the synergistic effect was derived from the associated benefits proposed by Ayers and Walter [10]. The associated benefits were used to show that the emission reduction measures related to greenhouse gases, such as CO_2_, could reduce generation of other pollutants. Thereafter, the IPCC [11] formally put forward the concept of synergy in their third assessment report. Synergy refers to the phenomenon in which implementation of a certain pollutant emission reduction measure generates other environmental benefits while achieving pollutant emission reduction. The existing research primarily focuses on the synergy effect of carbon abatement policies on emission reduction of air pollutants, the synergy effect of environmental governance on carbon abatement and climate change, countermeasures for reducing pollution and carbon, and evaluation of their synergy effects, etc.

Climate warming has attracted academic attention, and various scholars have discussed synergy effects related to relevant carbon abatement policies on reduction of atmospheric pollutants. Relevant policy research covers international climate agreements [12], construction of a carbon trading market [13], carbon tax [14], vegetation restoration [15], energy policy [16], etc. The research regarding synergy includes improvement of air quality [17], residents’ health [18], the ecological environment [19], energy security, and economic aspects [20], etc. For example, Ramanathan and Xu confirmed that the energy sector carried out large-scale decarbonization according to the requirements of the Copenhagen Agreement, which reduced carbon emissions and decreased emissions of ozone, methane, and black carbon (BC) [21]. Vandyck et al. stated that, if the emission reduction commitments of the Paris Agreement could be met, air quality could be greatly improved globally and the synergistic effects of emission reduction could offset expenditure from climate policies [22]. By studying ASEAN countries, Anser et al. revealed that financing of low-carbon energy could reduce air pollution and improve the environment [23]. Yan et al. argued that the low-carbon city pilot (LCCP) policy could curb haze pollution by improving industry structure, boosting technical innovation, and decreasing energy consumption [24]. Jin et al. empirically analyzed the synergistic effect of low-carbon technological innovation on haze pollution in 30 provinces of China from 2006 to 2018, showing that low-carbon technological innovation inhibited air pollution and produced a synergistic effect of positive externalities between regions in the long run [25].

Environmental governance studies demonstrate that reducing discharge of air pollutants, solid waste, and wastewater pollutants is a good way to attain environmental governance, and reducing emissions of pollutants can bring about such synergistic effects as carbon emission reduction and climate change. Okorn et al. verified synergy of pollutant emission reduction by simultaneously monitoring air pollutant emissions and CO_2_ emissions in a community in Los Angeles [26]. Peng Wen et al. conducted a scenario simulation focused on the industrial policy of reducing pollution and carbon. They claimed that a 10% increase in terms of energy efficiency of industrial sectors would have huge benefits for China’s air quality, public health, and climate [27]. Wang et al. studied synergy effects related to decreasing carbon discharge and air pollution during execution of the pilot policy for low-carbon cities. They found that the policy of low-carbon cities significantly reduced concentrations of CO_2_ and PM_2.5_, and the co-control effect of decreasing carbon discharge and air pollution was remarkable [28].

In recent years, most scholars have introduced multiple indicators to discuss the synergistic effect of PRCA, and measures to improve PRCA fall into three categories: technical improvement [29], structural adjustment [30], and supervision and management [31]. Using a DEA window analysis, Zou gauged the input and output efficiency of controlling industrial pollution, revealing that China’s overall efficiency in controlling industrial pollution exhibited a downward tendency in the study period. They posited that improving efficiency in controlling industrial pollution was essential for degrading pollution discharge and attaining carbon neutralization and carbon peak [32]. Anderson assessed the influence of green infrastructure productive applications on air pollution and CO_2_ concentration across different agricultural forms of production, showing that productive application of green infrastructure was as beneficial as nonproductive applications in reducing ozone, nitrogen dioxide, and CO_2_ concentration [33]. By comparing the regional differences in PRCA collaborative governance in China’s three leading urban agglomerations, Di Qianbin et al. revealed that the internal differences in degree of PRCA collaborative governance in Hebei, Tianjin, and Beijing gradually decreased and the internal differences in the Yangtze River Delta remained essentially stable, while the internal differences in the Pearl River Delta fluctuated greatly [34].

Urban agglomerations are the chief drivers of regional economic development and the places with the highest concentrations of carbon discharge and air pollution [35]. With increasing mobility of the economy, resources, and other elements in the regional urban network, isolated cities are gradually evolving into closely linked urban agglomerations. However, as urban agglomerations develop, they also face multiple pressures, such as addressing climate change, ecological conservation, and economic growth [36]. From the perspective of urban agglomerations, studying, planning, and promoting PRCA collaborative governance in urban agglomerations may represent significant avenues through which to promote overall green conversion in economic and social growth and thoroughly improve the quality of the ecological environment. As an important center of the chemical, energy, and production industries, the Yellow River Basin has a vulnerable climate with fragile ecology and concentrated carbon emissions and pollution. The imbalance and state development are inadequate. It is urgent to steadily move towards carbon abatement, pollution reduction, and high-quality development in the basin by shifting the role of urban agglomerations from point to area. At present, various elements need to be studied thoroughly, such as the synergy effect of PRCA in urban agglomerations in the basin, the features of its spatiotemporal evolution pattern, and the factors that affect effectiveness of regional coordinated governance of urban agglomeration for reducing pollution and carbon. Therefore, in this study, we constructed an evaluation index system for PRCA collaborative governance in urban agglomerations using the dimensions of environmental pollution, environmental governance, resource utilization, and economic development. In addition, we explored the order degree and overall synergy degree of PRCA collaborative governance in urban agglomerations in the basin from 2006 to 2020 using models such as synergy of composite system. Moreover, we revealed regional variances in PRCA collaborative governance degree using the Gini coefficient and Theil index. On this basis, the paper classifies the basin’s urban agglomerations into regional collaborative governance groups by dividing the governance boundaries and using other methods in order to explore the factors affecting PRCA collaborative governance therein. We propose corresponding policy recommendations with a view to providing a reference with which to optimize PRCA collaborative governance in the urban agglomerations in the basin. This study provides an empirical reference for formulating differentiated collaborative governance strategies for PRCA, comprehensive green and low-carbon economic and social transformation programs, and high-quality green development paths in urban agglomerations, which is of certain theoretical and practical significance.

The main contributions of this paper are as follows: first, regarding investigation of level of inter-regional PRCA collaborative governance, the existing studies are mainly conducted from a single dimension, while this study establishes the assessment index system of PRCA collaborative governance in four dimensions. Second, the paper reveals the spatiotemporal evolution features of PRCA collaborative governance level on urban agglomeration scale, measures PRCA collaborative governance degree of urban agglomerations using the correlation coefficient matrix and composite system synergy degree, and analyzes regional differences in PRCA collaborative governance of urban agglomerations by combining the Gini coefficient and Thiel index. Finally, this paper discusses the factors that affect PRCA collaborative governance in the basin’s urban agglomerations and explores the impact of different factors in urban agglomerations on PRCA collaborative governance.

The remaining sections are arranged as follows. Section 2 introduces a regional overview of the Yellow River Basin and establishes an assessment index system for PRCA collaborative governance. Section 3 presents and explains the research methods. Section 4 discusses the spatiotemporal evolution features of and regional differences in PRCA collaborative governance in the basin’s urban agglomerations. Section 5 explores the extent to which different affecting factors play a role in PRCA collaborative governance in the basin’s urban agglomerations. Finally, conclusions are offered in the final section.

## 2. Research Area, Research Framework, and Indicator System

### 2.1. Study Areas

**Figure 1:**
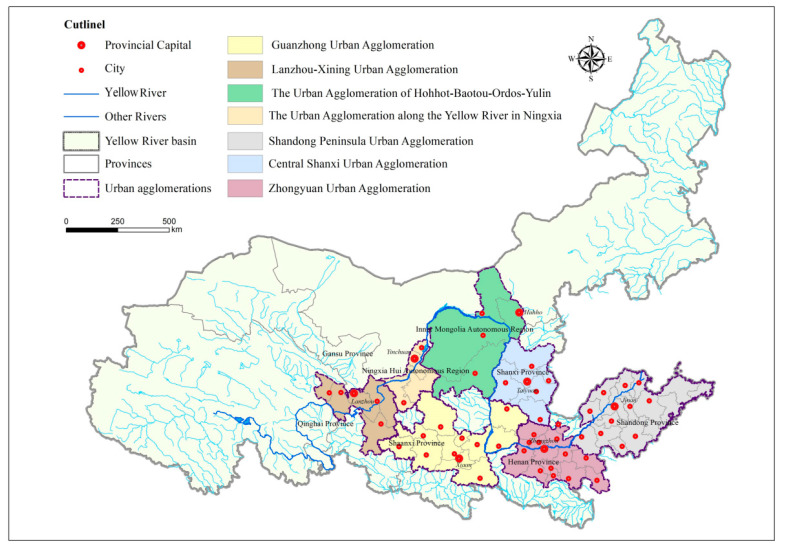
Research area.

The Yellow River Basin covers nine provinces, which contains three mature urban agglomerations and four emerging urban agglomerations, forming a “3 + 4” spatial organization pattern (as shown in Figure 1 and Table 1). From the dual perspectives of spatial pattern and historical laws, the basin serves as the center of the energy industry, basic production industry, chemical industry, and raw materials industry in China. It is characterized by its energy intensity, fragile ecology, climate vulnerability, high energy consumption, and CO_2_ emission intensity, which account for the emphasis on energy conservation and carbon reduction [37]. Moreover, affected by regional endowment and other factors, the relevant regions and provinces in the basin need to improve their economic connectivity, their quality of division and cooperation, and their level of coordinated growth. It is urgent to steadily promote collaboration between economic growth and ecological conservation by giving play to the important role of urban agglomeration from point to area [38].

### 2.2. PRCA Collaborative Governance Evolution Mechanism and Framework

PRCA collaborative governance is essentially the transformation of ecological environment governance from “pollution before governance” to “source prevention” and “source governance”. Guided by high-quality economic development, various measures are taken to promote the transformation and development of PRCA and expedite formation of resource-conserving and environment-friendly energy structures.

**Figure 2:**
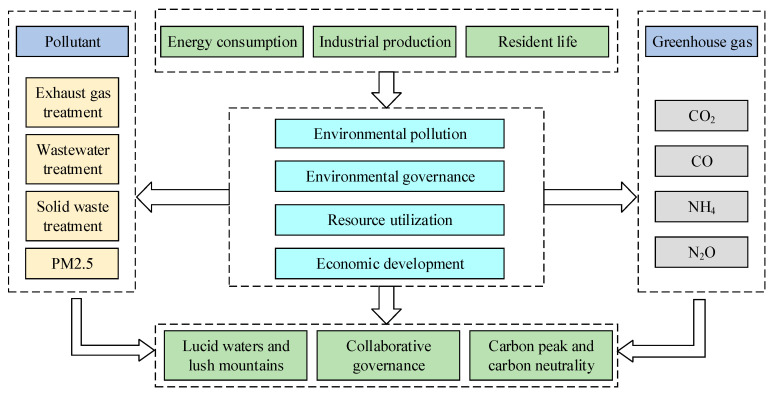
The PRCA collaborative governance evolution mechanism.

Figure 2 shows that environmental pollutant discharge and greenhouse gas emissions in the basin’s urban agglomerations mainly originate from the existing industrial structures, i.e., the heavy chemical industries and energy structure related to coal, human activities, such as energy consumption, industrial production, and the residents’ lives. Environmental pollutants and greenhouse gases have a certain degree of homology, and PRCA collaborative governance should be conducted according to the same frequency, effect, and path. Administrative coordination, industrial agglomeration, and close economic ties of urban agglomeration cities promote PRCA in urban agglomerations, and it is more convenient for urban agglomerations to make overall arrangements concerning environmental pollution and governance, energy and industrial structure optimization, ecological environment coordination, and comprehensive utilization of resources. With reference to relevant research, this paper presents the idea that PRCA collaborative governance system in urban agglomerations includes subsystems of environmental pollution, environmental governance, resource utilization, and economic development. Among them, the environmental pollution subsystem weighs the pollution status during the process of urban agglomeration development, the environmental governance subsystem measures PRCA level, the resource utilization subsystem assesses resource utilization efficiency in the process of reducing pollution and carbon, and the economic development subsystem evaluates economic support in the process of reducing pollution and carbon.

### 2.3. Index System for Evaluating PRCA Collaborative Governance

Taking PRCA collaborative governance of urban agglomerations as the overall system, PRCA collaborative governance of cities as the subsystem, and the four aspects of environmental pollution, environmental governance, resource utilization, and economic development as the order parameters, this paper gradually screened, optimized, and tested the relevant order variables. With reference to relevant research [39,40,41,42,43], an evaluation index system for PRCA collaborative governance synergy in the basin’s urban agglomerations was established, including four order parameters and eighteen order variables (as shown in Table 2).

(1) Environmental pollution. In full consideration of the comprehensiveness and complexity of environmental pollution, we selected five indicators as order variables to gauge atmospheric and water pollution: discharge of industrial wastewater, discharge of industrial sulfur dioxide (SO), total discharge of smoke (dust), CO_2_ discharge, and annual average PM_2.5_ concentration.

(2) Environmental governance. Environmental governance is the core component of urban sustainable development. Representative indicators were chosen from the angles of water, solid waste, and garbage treatment, and rate of industrial solid waste integrated processing, centralized processing rate of sewage treatment plants, rate of innoxious disposal of urban garbage, and disposal rate of urban domestic sewage were used to reflect the emission control levels of various environmental pollutants.

(3) Resource utilization. In addition to economic growth, intensive use of resources is also a great concern. Total energy consumption was introduced to reflect energy use, coal consumption was used to reflect energy consumption structure, and total water supply was used to reflect the control level of regional water resource utilization in the study.

(4) Economic development. Economic development is substantially manifested in economic aggregate expansion and improvement of developmental quality. In this study, regional GDP was adopted to reflect total economic output, GDP growth rate was applied to assess quality of urban wealth creation, per capita disposable income and social consumable total retail sales were used to reflect national consumption capacity, direction of economic development was evaluated by amount of regional social fixed assets investment, and added value ratio of tertiary industry in GDP was employed to represent the optimized structure of the urban economic structure.

## 3. Methodology

### 3.1. Index Weight Measurement Method

The correlation coefficient matrix was introduced to measure weight so as to avoid the influence of subjective factors [44]. Supposing there are n indexes in the index system, their correlation matrix x is

(1) $$x=\left[\begin{array}{ccc} a_{11} & \cdots & a_{1n}\\ \vdots & \ddots & \vdots \\ a_{n1} & \cdots & a_{nn} \end{array}\right],x_{i}=\sum_{j=1}^{n}\left|a_{ij}\right|-1,i=j=1,2\cdots ,n$$

where $a_{ij}$ refers to index i of city j and $x_{i}$ indicates the total impact of index i on indexes n−1. The larger the $x_{i}$ value is, the more important $x_{i}$ is to the whole index system and the greater the weight should be given. Therefore, when we normalize $x_{i}$, the weight $\theta_{i}$ of each index can be obtained:

(2) $$\theta_{i}=\frac{x_{i}}{\sum_{i=1}^{n}x_{i}},i=1,2,\cdots ,n$$

### 3.2. The Composite System Synergy Model

#### 3.2.1. Order Degree of Subsystem

The order parameter of PRCA collaborative governance is $T_{w}=(T_{w1},T_{w2},\ldots ,T_{\mathrm{wm}}),m\geq 1$. If the values of $T_{w1},T_{w2},\ldots ,T_{\mathrm{wt}}$ are larger, the system order degree will be higher. If the values are lower, the system order degree will be lower. Then, the order degree of the order variable components of the subsystem was calculated using the following formula [45,46]:

(3) $$U_{w}\left(T_{wk}\right)=\{\begin{array}{c} \frac{T_{\mathrm{wk}}-b_{wk}}{a_{wk}-b_{wk}},\;\;\;k\in [1,t]\\ \frac{a_{wk}-T_{wk}}{a_{wk}-b_{wk}},\;\;\;k\in [t+1,m] \end{array}$$

where $U_{w}\left(T_{wk}\right)$ indicates the order degree of order parameters. When $U_{w}\left(T_{wk}\right)\in \left[0,1\right]$, the greater the value is, the better the PRCA collaborative governance of the subsystem is. $T_{\mathrm{wk}}\in \left[a_{wk},b_{wk}\right],a_{wk},b_{wk}$ express the minimum and maximum values, respectively, of the first order variable of the PRCA collaborative governance of the subsystem. The order degree of urban sub-governance system was obtained using the following equation [47]:

(4) $$U_{w}\left(T_{w}\right)=\sqrt[{m}]{\prod_{k=1}^{m}U_{w}\left(T_{wk}\right)}\times P_{w}$$

where $U_{w}\left(T_{w}\right)$ indicates the order degree of the subsystem and $P_{w}$ represents the weight coefficient, $P_{w}$ ≥ 0 and $\sum_{w=1}^{n}P_{w}=1$.

#### 3.2.2. The Composite System Synergy

Given the research period from initial moment $t_{0}$ to moment $t_{1}$, the order degree of each urban governance subsystem is $U_{w}^{0}\left(T_{w}\right),U_{w}^{1}\left(T_{w}\right)$, and the overall synergy degree of the PRCA composite collaborative governance system from moment $t_{0}$ to moment $t_{1}$ is as follows [48]:

(5) $$\begin{array}{l} F=\beta \sum_{w=1}^{n}\gamma_{w}\left[\left|U_{w}^{1}\left(T_{w}\right)-U_{w}^{0}\left(T_{w}\right)\right|\right]\\ \beta =\frac{\min\left[U_{w}^{1}\left(T_{w}\right)-U_{w}^{0}\left(T_{w}\right)\right]}{\left|\min\left[U_{w}^{1}\left(T_{w}\right)-U_{w}^{0}\left(T_{w}\right)\right]\right|} \end{array}$$

where F denotes synergy degree of composite systems, with higher values indicating a higher synergy degree. β represents stability of the system synergy degree; when β < 0, the system as a whole is in an unstable or noncollaborative state; when β and >0, the system is in a positive collaborative state. $\gamma_{w}$ denotes the weight coefficient. Composite system synergy was numerically calculated relative to the base period of 2006.

### 3.3. Measurement of Regional Differences in PRCA Collaborative Governance

The Gini coefficient (Gini) and Theil index (TB) were employed to analyze the PRCA composite system synergy difference of the urban agglomerations, which were calculated using the following equations [49]:

(6) $$Gini=\frac{-\left(s+1\right)}{s}+\frac{2}{s^{2}y}\sum my_{m}$$

(7) $$TB=\frac{1}{s}\sum_{i=1}\frac{y_{m}}{u}\ln\frac{\bar{y}}{u}$$

where s denotes sample numbers, $y_{m}$ denotes the index value of the m sample, and ${\bar{y}}_{m}$ denotes the mean value of all sample index values. In this paper, TB is further decomposed into gaps within an urban agglomeration and gaps between urban agglomerations. The decomposition method is shown as follows [50]:

(8) $$TB=T_{BL}+T_{BD}=\sum_{q}^{3}f_{q}\ln\frac{u_{q}}{u}+\sum_{q}^{3}f_{q}\frac{u_{q}}{u}\ln\frac{u_{q}}{u}$$

where $u_{q}$ represents the PRCA synergy value in the urban agglomeration q, u denotes the synergy mean value of the collaborative governance of PRCA, $f_{q}$ denotes the ratio of the number of cities to the total number of cities in the urban agglomeration q, $T_{BL}$ represents intragroup differences, and $T_{BD}$ denotes the difference between groups.

### 3.4. Data Collection

The study selected relevant data concerning urban agglomerations in the Yellow River Basin from 2006 to 2020. Most of the data were selected from *CHINA CITY STATISTICAL YEAR BOOK*, *CHINA INDUSTRY STATISTICAL YEAR BOOK*, *CHINA STATISTICAL YEARBOOK OF THE TERITARY INDUSTRY*, *CHINA STATISTICAL YEARBOOK ON ENVIRONMENT,* and *CHINA ENERGY YEARBOOK*. In addition, the relevant data on carbon emissions were obtained from *China Emission Accounts and Datasets*.

## 4. Analysis of Spatiotemporal Evolution Features

### 4.1. Assessment of Order Degree and Synergy Degree of PRCA Collaborative Governance

**Figure 3:**
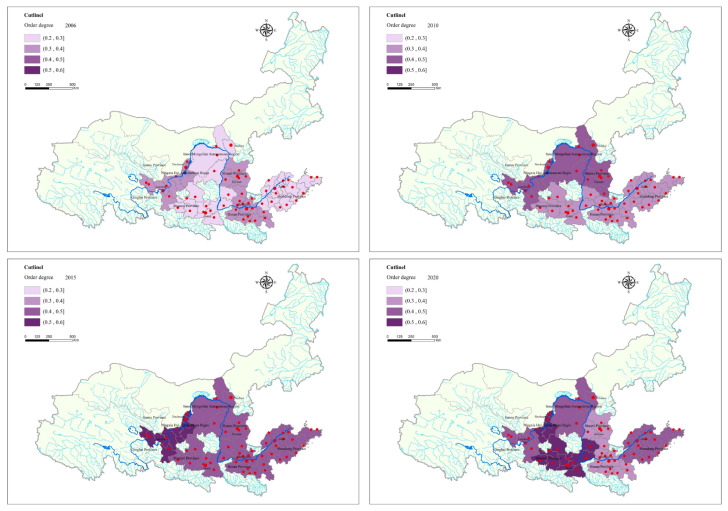
The PRCA collaborative governance order degree in the basin’s urban agglomerations in the years 2006, 2010, 2015, and 2020.

According to the mode and characteristics of PRCA collaborative governance in the urban agglomerations, in order to better reflect the order parameters and overall degree of order of the subsystems, we assessed the weight of each order parameter using the correlation coefficient matrix. Considering the different dimensions of each order parameter, the original data were processed in a dimensionless manner and introduced into Formula (3) to obtain the order degree of each order parameter. Then, the index weight determined by the correlation coefficient matrix method was applied in Formula (4) to obtain the order degree of PRCA governance system of the seven urban agglomerations from 2006 to 2020 (as shown in Figure 3). The order degree of PRCA collaborative governance in the seven urban agglomerations in the Yellow River Basin exhibited a significant growing trend, and there existed certain variances in the growth rate of the order degree in each urban agglomeration. This revealed the spatial evolution feature of being “high in the west and low in the east”. The synergy of the PRCA collaborative governance system increased constantly, and economic growth and eco-environmental preservation in the urban agglomerations accelerated.

**Figure 4:**
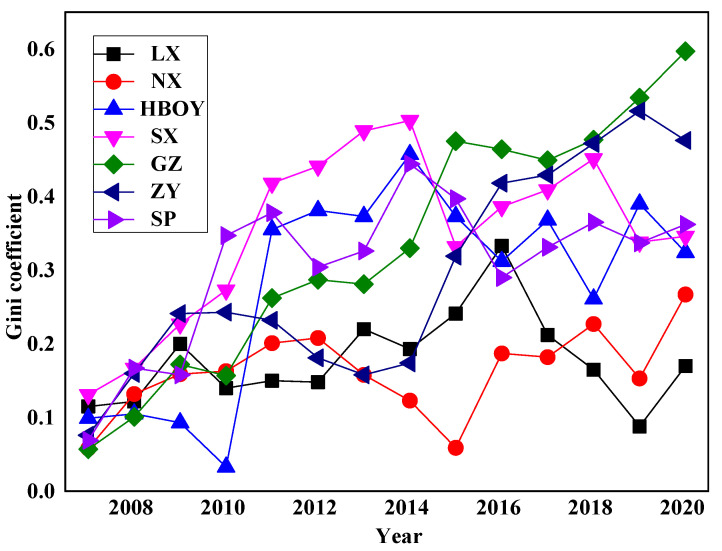
The PRCA collaborative governance synergy of urban agglomerations in the Yellow River Basin from 2006 to 2020.

In the Yellow River Basin, PRCA collaborative governance system of the seven urban agglomerations exhibited different characteristics related to developmental stage. With the view of reflecting the status of overall PRCA collaborative governance accurately, we took the year 2006 as the base research period and the total value index of each subsystem as the variables. According to Formula (5), we obtained the PRCA collaborative governance synergy of the urban agglomerations from 2007–2020 (as shown in Figure 4). With regard to the change trend in synergy degree, GZ and ZY grew faster, SP, HBOY, and SX exhibited small overall fluctuations and were in a relatively stable state, and LX and NX were the slowest. In general, the seven basin urban agglomerations exhibited low collaborative governance synergy in terms of PRCA, which indicates that internal coordination in the urban agglomerations had not yet formed and that mechanisms functioning on a deeper level had yet to be established. The overall synergy degree fluctuated greatly, and the system was in a disordered and unsteady state, revealing that there was certain practical resistance to PRCA collaborative governance in the seven urban agglomerations in the basin. GZ, ZY, and SP were characterized by an early start, rapid development, obvious regional advantages, great integral capabilities, and unique ecological resources, which accounts for the high PRCA collaborative governance synergy in these three urban agglomerations. PRCA collaborative governance synergy in HBOY and SX first exhibited an ascending trend and then a descending trend. The ecological environment stress effect caused by economic growth relying on coal and other resources restrained their collaborative governance synergy to a certain degree. Industrial growth of these two urban agglomerations rests heavily on resources, and economic growth depends on resource consumption. It is difficult to adjust the structure of coal-dominant energy consumption, and it is likely that they will fall into the “resource curse”. Since 2015, with the development of urbanization, the ecological environment has been sacrificed, the intensity of pollutant emissions has remained high, and, thus, the air quality is difficult to improve in any fundamental sense. LX and NX exhibited relatively low PRCA collaborative governance synergy, which is attributed to the position of ecological function protection areas in western China, strong constraints related to environmental policies and systems, and high dependence on resources and ecosystems for economic development. However, as a result of the impact of national industrial structure strategy adjustments, the advantages of the traditional energy mining industry are disappearing, and the job of green transformation and pollution control is challenging. Moreover, there are other problems in the region, such as the poor level of industrial structure and weak innovation driving force, which make it difficult for them to improve in terms of PRCA collaborative governance synergy.

### 4.2. Regional Differences in PRCA Collaborative Governance Synergy

In accordance with the Gini coefficient result for PRCA collaborative governance synergy from 2007 to 2020 (as shown in Table 3), the PRCA collaborative governance synergy of the seven basin urban agglomerations was significantly different. The internal difference in synergy of LX gradually decreased, and, from 2007 to 2020, the Gini coefficient decreased from 0.249 to 0.045, first showing an increasing trend with fluctuations and then a decreasing trend. The internal difference in collaborative governance synergy in NX and GZ was basically stable, with a small fluctuation range. The internal difference in collaborative governance synergy in HBOY, SX, SP, and ZY gradually decreased, and the Gini coefficients decreased from 0.290, 0.306, 0.213, and 0.296 to 0.157, 0.120, 0.147, and 0.149, respectively, from 2007 to 2020. The internal synergy difference for each urban agglomeration decreased. In general, the cities in the basin’s urban agglomerations were at various developmental stages, and there was great difference in environmental pollution conditions, environmental governance capacities, and economic development levels in these cities, leading to significant pressure on PRCA collaborative governance.

Table 4 shows the results calculated using Formulas (7) and (8). During the study period, the Theil index for PRCA collaborative governance synergy of the urban agglomerations in the basin exhibited a downward trend in which the PRCA collaborative governance synergy remained essentially stable in NX and GZ and continued a declining trend in LX, HBOY, SX, ZY, and SP. As growing attention has been paid to the ecological security in the Yellow River Basin, optimal allocation of resources has led to orderly flow of factors and improved ecological environment. PRCA collaborative governance synergy in LX, HBOY, SX, ZY, and SP has gradually decreased. However, industrial development of NX and GZ has been highly resource-dependent; it is difficult to adjust the coal-based energy consumption structure, and the development mode of high energy consumption and high pollution is still prevailing. Utilizing rich resources to develop the economy also inevitably results in great damage and pollution to the regional ecological environment in NX and GZ. In addition, rapid accumulation of population and industry makes resource and environmental load increase too fast, which has led to doubling of pressure on the ecological environment. The gaps between urban agglomerations and within urban agglomerations were relatively large. Generally, regional intragroup contributions, for the most part, accounted for regional differences.

## 5. Analysis of Affecting Factors

### 5.1. Model Specification

Based on research concerning the factors influencing PRCA collaborative governance synergy, we analyzed the influence of the variances among these influencing factors on PRCA collaborative governance synergy within the group. The model was established as follows:

(9) $$SD_{it}=\beta_{0}+\sum_{k=1}^{n}\beta_{k}\frac{\sigma_{X_{it}^{k}}}{X_{it}^{k}}+\mu_{i}+\varepsilon_{it}$$

where i refers to governance group, t represents year, $SD_{it}$ indicates PRCA collaborative governance synergy of i governance group in t year, $\beta_{0}$ denotes constant term, $\beta_{k}$ denotes coefficient of influencing factors, $\sigma_{X_{it}^{k}}$ is standard deviation of influencing factors within governance group, $X_{it}^{k}$ is mean value of influencing factors in governance group, $\mu_{i}$ represents individual fixation effects, and $\varepsilon_{it}$ denotes stochastic error terms.

### 5.2. Variable Selection

Based on Model (9), PRCA synergy degree (SD) expresses the variables explained in this study. When selecting explanatory variables, taking the existing literature into consideration, we extracted six indicators to probe the factors that affect PRCA synergy degree (as shown in Table 5): industrial structure [51,52,53,54,55], environmental regulation [56,57], economic growth [58,59,60,61,62], energy consumption [63,64,65], greening construction [66,67], and opening-up [68,69].

(1) Industrial structure variance. Industry is an essential driving force for economic and social development in the basin’s urban agglomerations and represents the main source of local air pollutants and carbon emissions. At present, the industrial structure of the basin’s urban agglomerations is composed of the heavy chemical and energy industries, which causes obvious problems, such as extensive industrial development and a low proportion of technology-intensive industries. Therefore, the proportion of secondary industry to GDP was employed to reflect the industrial structure, and the relative dispersion of the ratio of secondary industry to GDP was used to express regional differences in terms of industrial structure in this paper.

(2) Environmental regulation variance. From the perspective of public economics, as a public product with strong positive externality, environment is essentially a public service provided by the government. Environmental governance investment comes from government subsidies, enterprise self-financing, and bank loans, but it is ultimately affected by work of local governments. However, gravity of environmental pollution in the basin’s urban agglomerations is disproportionate to environmental protection investment. Moreover, there is a certain deviation that makes environmental governance investment fail to function as it should, i.e., to improve the environment. Therefore, this paper uses environmental governance investment to characterize environmental regulation and introduces relative dispersion of environmental governance investment to reflect the variances in environmental regulation between regions.

(3) Economic growth variance. Improvement in economic development tends to impact regional industrial structure, consumption structure, and other aspects. On the one hand, residents in areas with low economic development are more willing to trade environment for growth. On the other hand, in order to gain political support, local governments interfere in implementation of environmental policies in exchange for local economic growth. Accordingly, variance in economic development can affect the regional synergy of environmental governance. Therefore, in this paper, GDP per capita is employed to represent economic growth degree, and relative dispersion of GDP per capita is applied to reflect variances in economic growth between regions.

(4) Energy consumption variance. Generally, in regions where energy is produced from fossil fuels, high economic growth means more energy consumption, which is liable to generate more air pollution and carbon discharge. To reach the target of PRCA collaborative governance, the basin’s urban agglomerations need to control total energy consumption and reduce lagging capacity as increase in energy consumption variances between regions has an impact on PRCA collaborative governance. Therefore, this paper uses energy consumption intensity to express energy consumption and uses relative dispersion of energy consumption intensity to reflect variances in energy consumption among regions.

(5) Greening construction variance. A higher green coverage rate is more conducive to air purification and tends to promote the PRCA effect. Urban agglomerations in the Yellow River Basin should guarantee that the eco-environmental area will not decrease and strengthen eco-space protection. The increase in regional variances in greening construction have an impact on PRCA collaborative governance.

(6) Opening-up variance. With adjustments in regional cooperation of the global value chain, the regions and provinces in the basin are likely to become the key locations in which China participates in international cooperation and receives direct foreign investment in the future. It is unclear whether the pollution haven effect or pollution halo effect influence variances in degree of regional opening-up in PRCA collaborative governance. Therefore, this paper uses the scale of actual foreign capital utilization to characterize opening-up and uses the relative dispersion degree of the actual foreign capital utilization scale to reflect opening-up variances between regions.

### 5.3. Analysis of Empirical Results

According to Formula (9), the study employed Stata16 software to carry out regression analysis on the influencing factors of synergy degree of PRCA in the urban agglomerations in the Yellow River Basin. To guarantee that the model specification is objective, we first verified the variance expansion coefficient (VIF) for each explanatory variable. The values were all lower than 10, so it can be determined that there was not any multicollinearity among the explanatory variables. Second, the Hausman test was applied and the original hypothesis, i.e., in support of the stochastic effect model, was rejected. Thus, the regression model for the fixed effect panel was selected. Finally, the modified heteroscedasticity and autocorrelation *xtscc* were used for estimation (as shown in Table 6).

The estimated coefficient of industrial structure variance was significantly positive on the level of 1%, which, to some extent, shows that the variance in industrial structure was conducive to PRCA collaborative governance. For a long time, heavy chemical industries have dominated the Yellow River Basin, such as the petroleum processing industry, the nonferrous metal smelting industry, and the mining industry. Certain cities in SX have been manipulated by resource-intensive industries; thus, strategic emerging industries and modern service industries have developed relatively slowly, and industrial development and economic growth have negatively impacted the ecological environment. However, the more concentrated the polluting industries are in intensive industrial clusters, the more cautious the implementation of strict environmental management policies. Thus, maintaining industrial structure variance in urban agglomerations tends to positively influence PRCA collaborative governance.

The estimated coefficient for variance in environmental regulation was significantly positive on the 1% and 5% levels. Considering that current environmental governance investment has different effects on environmental improvement in various regions, certain regions have invested more in environmental governance, but the effects are not obvious. Each urban agglomeration should actively guide a shift in governance investment structure according to its own characteristics, adopt differentiated investment modes, further strengthen targeted governance of pollution sources, improve the investment effect of industrial pollution source governance, and strive for industrial transformation.

The estimated coefficient of variance in economic growth was significantly negative on the 1% and 5% levels, which demonstrates that variance in economic growth significantly restrains synergy of regional PRCA governance. This shows that, the greater the variance in per capita GDP among regions, the more unfavorable it is for PRCA collaborative governance. In other words, the greater the variance in per capita GDP among regions, the more difficult it is for PRCA collaborative governance.

The negative coefficient of estimation of energy consumption variance shows that energy consumption does not significantly inhibit PRCA collaborative governance synergy. Although the basin’s urban agglomerations currently prefer diversified development strategies based on efficiency and clean energy, the industrial structures of most cities in the basin are still in the order of secondary–primary–tertiary industries, in which traditional high-energy consumption industries occupy a large part and prevent the industrial structure from being effectively adjusted. Therefore, advantages and benefits of optimization and upgrading the energy structure for regional PRCA may not be effectively observed in the short term, so actively adjusting the industrial structure, eliminating outdated production capacities and processes, and optimizing industrial layout and process structures are particularly important to improve PRCA collaborative governance.

The estimated coefficient of variance in greening construction was negative, which did not significantly inhibit PRCA collaborative governance synergy. The better the degree of urban agglomeration greening construction, the greater the improvement in urban air environment quality and the better residents’ physical and mental health. In order to achieve efficient cooperation as regards PRCA among the basin’s urban agglomerations, the differences in social growth dimensions, such as regional greening construction, need to be constantly narrowed.

The estimated coefficient of variance in opening-up was negative, which did not significantly inhibit PRCA collaborative governance synergy. Moreover, the possible existence of a pollution haven or pollution halo could not be confirmed nor could its potential effects be assessed. The opening-up economic growth in the basin was extremely uneven, with a large gap in overall level of opening-up. In addition, the transportation conditions on the Yellow River are limited, and the overall economic ties between cities in the basin are very weak, so there is little close regional economic and trade coordination. For the sake of attracting foreign capital, local governments tend to use loosening environmental regulations as bait, which may generate a “Race to the Bottom” effect as regards environmental regulation. This also shows that there is still some room for improvement in the structure and efficiency of domestic and foreign investment in the Yellow River Basin.

### 5.4. Robustness Test

Considering that the factors affecting PRCA collaborative governance synergy degree have a certain lag, a robustness test was conducted on the explanatory variables with a one-period lag (as shown in Table 7).

## 6. Conclusions

In this paper, we construct an evaluation index system for PRCA collaborative governance in urban agglomerations from the dimensions of environmental pollution, environmental governance, resource utilization, and economic development. In addition, we explore the order degree and synergy degree of PRCA collaborative governance in the basin’s urban agglomerations from 2006 to 2020 by employing the composite system synergy model. Moreover, we reveal regional differences in PRCA collaborative governance synergy using the Gini coefficient and Theil index and then explore the factors that influence PRCA collaborative governance of the basin’s urban agglomeration. The findings are as follows:

First, the order degree of PRCA collaborative governance in the seven basin urban agglomerations exhibited a significant growth trend, increasing at a rapid rate, while the synergy degree of PRCA collaborative governance was relatively low, exhibiting a slow upward trend. Second, the internal variances in PRCA collaborative governance synergy gradually decreased in LX, HBOY, SX, ZY, and SP, and the internal differences remained essentially stable in GZ and NX. Finally, the regional variances in environmental regulation and industrial structure had significant positive influences on PRCA collaborative governance in the urban agglomerations, the economic growth variances had significant inhibiting effects, and the variances in energy consumption, greening construction, and opening-up negatively affected collaborative governance; however, the effect was not significant and the conclusion remained unchanged after robustness testing.

On account of the above findings, to further promote PRCA collaborative governance in the basin’s urban agglomerations, it is of great importance to accelerate industrial structure upgrading, strengthen regional cooperation, and reduce regional differences. Specific recommendations are offered in the following:

(1) The urban agglomerations in the basin should accelerate industrial structure upgrading and formulate differentiated collaborative strategies for PRCA. In accordance with their developmental stages, natural conditions, and resource endowment structures, each urban agglomeration should increase proportion of tertiary industry, actively advance high-tech industries (such as intelligent manufacturing, photovoltaic energy storage, new energy vehicles, bioenergy power generation, etc.), appropriately reduce proportion of secondary industries based on coal, petroleum, chemicals, and promote green and low-carbon transformation of the aforementioned key industries. They should increase pollution reduction and carbon abatement intensity and focus on energy structure adjustment, energy efficiency improvement, industrial structure transformation and upgrading, and multipollutant coordinated control.

(2) The urban agglomerations in the basin should strengthen regional cooperation to achieve PRCA collaborative governance among cities and industries. PRCA is a shared responsibility and regional cooperation is an important path to promote PRCA collaborative governance. They should use industrial developmental status and regional advantages of each urban agglomeration to promote industrial transfer and technology flow from developed regions to underdeveloped regions so as to attain industrial layout optimization among regions and PRCA collaborative governance. They should push development of urban regional integration, accelerate interconnection between urban infrastructure and spatial design of urban agglomerations, economic circles, and metropolitan areas, reduce administrative barriers and transaction costs, and further facilitate free flow of market elements. While increasing investment in environmental regulation in urban agglomerations, they should avoid the “Race to the Bottom” effect caused by excessive variances in environmental regulation intensity and attempt to establish a cross-regional market-oriented platform for pollution control. Furthermore, they should strengthen enthusiasm and commonality in terms of participating in PRCA collaborative governance so that they can improve the environmental governance capacity and cooperation performance in the basin.

(3) The urban agglomerations in the basin should comply with the trend of regional coordinated development and aim to constantly reduce regional differences. Taking the opportunity to construct a unified market, they should promote a new development pattern that is dominated by large-scale domestic circulation and is mutually promoted by domestic and international circulation. First, they should strengthen green technology by opening-up and adopt multiple paths, such as technology introduction, imitation, and learning, and independent innovation. Second, they should take the initiative to apply certain carbon abatement measures, such as volunteer domestic industrial structure transformation and energy system change, and encourage carbon abatement in terminal sectors, with the view of advancing the domestic eco-environment and raising the environmental threshold for foreign investment. Finally, they should accelerate implementation of environmental tax policies, formulate strict technology- and industry-access mechanisms, curb excessive supply of high-energy-consumption- and highly polluting technologies by foreign-funded enterprises, and effectively promote environmental technology spillover as a result of direct foreign investment.

Although we have conducted much work on the research subject, there is still room for improvement in future research. This paper only discusses spatiotemporal evolution features and factors that affect PRCA collaborative governance in the basin’s urban agglomerations. In the future, we will consider measuring the urban agglomeration’s green productivity in the Yellow River Basin under the constraint of PRCA. In addition, we will use the partial differential method of the spatial regression model to test and decompose the factors and spillover effect of green productivity in the urban agglomerations.

## Figures and Tables

**Table 1:** Overview of the urban agglomerations in the Yellow River basin.

Watershed	Urban Agglomerations	Function Orientation
Upper reaches	Lanzhou–Xining Urban Agglomeration (LX)	Strategic support for safeguarding national ecological security and the principal growth pole for supporting Northwest China development
	The Urban Agglomeration along the Yellow River in Ningxia (NX)	Important energy, chemical, and new material bases in China, and demonstration areas where man and nature develop in harmony in Northwest China
	The Urban Agglomeration of Hohhot–Baotou–Ordos–Yulin (HBOY)	The national high-end energy and chemical industry base, the strategic pivot of opening to the north and west, and ecological civilization cooperation and co-construction zone in Northwest China
Middle reaches	Central Shanxi Urban Agglomeration (SX)	The foundations of important national energy and advanced manufacturing, and the core display zone of integrated reform of national resource-based economic transformation of Shanxi
	Guanzhong Urban Agglomeration (GZ)	The strategic pivot of opening to the west, the valued growth pole that leads Northwest China to develop, and the pioneer area for inland ecological civilization construction
	Zhongyuan Urban Agglomeration (ZY)	The new growth pole of economic development, the foundation of the important industry of high-level manufacturing and industry of modern service, and demonstration zone of green eco-development
Lower reaches	Shandong Peninsula Urban Agglomeration (SP)	The demonstration zone of China’s blue economy and the efficient ecological economic zone

**Table 2:** Index system for evaluating PRCA collaborative governance of urban agglomeration in the Yellow River Basin.

Order Parameters	Order Variables	Orientations
Environmental pollution	industrial wastewater effluent	-
	industrial SO discharge	-
	industrial smoke (powder) emissions	-
	CO_2_ emissions	-
	annual average concentration of PM_2.5_	-
Environmental governance	the rate of industrial solid waste integrated processing	+
	innoxious disposal rate of urban garbage	+
	processing rate of urban domestic sewage	+
	centralized processing rate of sewage treatment plants	+
Resource utilization	total energy consumption	-
	coal consumption	-
	total water supply	-
Economic development	GDP	+
	GDP growth rate	+
	per capita disposable income	+
	total social consumable retail sales	+
	the amount of regional social fixed assets investment	+
	the proportion of added value of tertiary industry	+

**Table 3:** Gini coefficients of the PRCA collaborative governance synergy in urban agglomerations from 2007 to 2020.

	LX	NX	HBOY	SX	GZ	SP	ZY
2007	0.249	0.233	0.290	0.306	0.234	0.213	0.296
2008	0.093	0.088	0.181	0.216	0.206	0.231	0.200
2009	0.176	0.099	0.174	0.217	0.251	0.211	0.262
2010	0.106	0.102	0.219	0.156	0.133	0.171	0.204
2011	0.332	0.150	0.134	0.125	0.223	0.108	0.246
2012	0.242	0.142	0.241	0.214	0.156	0.119	0.204
2013	0.187	0.106	0.207	0.107	0.156	0.164	0.216
2014	0.130	0.132	0.133	0.096	0.175	0.160	0.237
2015	0.062	0.169	0.098	0.185	0.279	0.172	0.178
2016	0.112	0.174	0.218	0.111	0.189	0.095	0.194
2017	0.172	0.257	0.157	0.148	0.209	0.160	0.152
2018	0.138	0.144	0.236	0.151	0.197	0.121	0.153
2019	0.151	0.178	0.152	0.131	0.255	0.162	0.144
2020	0.045	0.204	0.157	0.120	0.277	0.147	0.149

**Table 4:** Theil index of the PRCA collaborative governance synergy of the urban agglomerations from 2007 to 2020 and decomposition.

Year	LX	NX	HBOY	SX	GZ	ZY	SP	Intragroup Difference	Intergroup Difference	Intragroup Contribution	Intergroup Contribution
2007	0.102	0.092	0.150	0.168	0.092	0.147	0.071	0.109	0.004	96.33%	3.67%
2008	0.015	0.016	0.057	0.073	0.067	0.066	0.085	0.072	0.293	19.78%	80.22%
2009	0.061	0.018	0.054	0.075	0.104	0.110	0.070	0.058	0.228	20.16%	79.84%
2010	0.018	0.019	0.088	0.042	0.028	0.065	0.046	0.054	0.134	28.83%	71.17%
2011	0.206	0.041	0.035	0.025	0.093	0.098	0.018	0.408	0.157	72.24%	27.76%
2012	0.099	0.033	0.126	0.104	0.040	0.068	0.025	0.350	0.033	91.33%	8.67%
2013	0.069	0.018	0.083	0.019	0.039	0.078	0.049	0.081	0.120	40.44%	59.56%
2014	0.030	0.030	0.036	0.019	0.051	0.088	0.050	0.077	0.317	19.52%	80.48%
2015	0.007	0.048	0.018	0.057	0.122	0.067	0.054	0.005	0.387	1.38%	98.62%
2016	0.023	0.050	0.081	0.022	0.065	0.065	0.015	0.241	0.121	66.61%	33.39%
2017	0.061	0.130	0.045	0.037	0.068	0.038	0.054	0.152	0.020	88.18%	11.82%
2018	0.033	0.037	0.100	0.040	0.063	0.038	0.026	0.182	0.010	94.91%	5.09%
2019	0.042	0.052	0.039	0.040	0.105	0.036	0.045	0.040	0.148	21.10%	78.90%
2020	0.004	0.070	0.040	0.028	0.127	0.037	0.038	0.183	0.339	34.98%	65.02%

**Table 5:** Variable descriptive statistics.

Variables	Abbreviations	Obs	Mean	Std.	Min	Max
Synergy degree	*SD*	105	0.2591	0.1384	0.0331	0.5975
Industrial structure variance	*Vind*	105	0.1850	0.0589	0.0513	0.3269
Environmental regulation variance	*Vipc*	105	0.6162	0.1929	0.1168	1.0865
Economic growth variance	*Vpgdp*	105	0.4218	0.0719	0.2687	0.5936
Energy consumption variance	*Venergy*	105	0.3213	0.0985	0.1440	0.5388
Greening construction variance	*Vgreen*	105	0.3085	0.6007	0.0162	3.3443
Opening-up variance	*Vfdi*	105	1.3629	0.6396	0.5369	2.9745

**Table 6:** Factors affecting PRCA collaborative governance.

	(1)	(2)	(3)	(4)	(5)	(6)
*Vind*	1.268 ***	1.425 ***	1.373 ***	1.415 ***	1.425 ***	1.434 ***
	(4.405)	(4.964)	(4.872)	(4.834)	(4.842)	(4.821)
*Vipc*		0.320 **	0.396 ***	0.406 ***	0.406 ***	0.411 ***
		(2.428)	(2.964)	(2.999)	(2.992)	(2.989)
*Vpgdp*			−0.599 **	−0.591 **	−0.562 **	−0.550 *
			(−2.174)	(−2.129)	(−1.992)	(−1.921)
*Venergy*				−0.101	−0.125	−0.102
				(−0.564)	(−0.679)	(−0.509)
*Vgreen*					−0.010	−0.010
					(−0.613)	(−0.648)
*Vfdi*						−0.012
						(−0.304)
*Cons*	0.025	−0.202	0.014	0.029	0.025	0.024
	(0.456)	(−1.888)	(0.097)	(0.196)	(0.171)	(0.163)
*N*	105	105	105	105	105	105
*R* ^2^	0.740	0.757	0.771	0.772	0.773	0.773

Note: The definitions of variable symbols are the same as those in Table 5; ***, **, and *, respectively, express significance at levels of 1%, 5%, and 10%.

**Table 7:** Test based on independent variables with one-period lag (L).

	(1)	(2)	(3)	(4)	(5)	(6)
*L.Vind*	1.231 ***	1.425 ***	1.373 ***	1.415 ***	1.425 ***	1.434 ***
	(3.930)	(4.410)	(4.264)	(4.241)	(4.242)	(4.257)
*L.Vipc*		0.315 **	0.371 **	0.380 **	0.378 **	0.385 **
		(2.022)	(2.320)	(2.345)	(2.361)	(2.389)
*L.Vpgdp*			−0.408	−0.402	−0.498	−0.468
			(−1.381)	(−1.355)	(−1.663)	(−1.537)
*L.Venergy*				−0.092	−0.099	−0.100
				(−0.449)	(−0.540)	(−0.498)
*L.Vgreen*					−0.010	−0.010
					(−0.613)	(−0.648)
*L.Vfdi*						−0.013
						(−0.372)
*Cons*	0.044	−0.182	−0.034	−0.018	0.006	0.004
	(0.744)	(−1.451)	(−0.206)	(−0.105)	(0.033)	(0.024)
*N*	98	98	98	98	98	98
*R^2^*	0.711	0.726	0.733	0.733	0.743	0.744

Note: The definitions of variable symbols are the same as those in Table 5; ***, **, respectively, represent significance at levels 1%, 5%.

## Data Availability

Readers can obtain the raw datasets used in this paper by themselves through the data sources described in Section 3, or by contacting the first author or the corresponding author.

## References

[1] Lee J.Y., Marotzke J., Bala G., Cao L., Corti S., Dunne J.P. (2021). Future Global Climate: Scenario-Based Projections and Near-Term Information.

[2] IEA (2022). World Energy Outlook 2022, International Energy Agency, Paris. License: CC BY 4.0 (report); CC BY NC SA 4.0 (Annex A). https://www.iea.org/reports/world-energy-outlook-2022.

[3] Wolf M.J., Emerson J.W., Esty D.C., de Sherbinin A., Wendling Z.A. (2022). Environmental Performance Index.

[4] Brągoszewska E., Mainka A. (2022). Impact of Different Air Pollutants (PM_10_, PM_2.5_, NO_2_, and Bacterial Aerosols) on COVID-19 Cases in Gliwice, Southern Poland. Int. J. Environ. Res. Public Health.

[5] Eum J., Kim H. (2021). Effects of Air Pollution on Assaults: Findings from South Korea. Sustainability.

[6] Fuller R., Landrigan P.J., Balakrishnan K., Bathan G., Bose-O’Reilly S., Brauer M., Caravanos J., Chiles T., Cohen A., Corra L. (2022). Pollution and health: A progress update. Lancet Planet. Health.

[7] Fu Y., Supriyadi A., Wang T., Wang L., Cirella G.T. (2020). Effects of Regional Innovation Capability on the Green Technology Efficiency of China’s Manufacturing Industry: Evidence from Listed Companies. Energies.

[8] Friedlingstein P., O’Sullivan M., Jones M.W., Andrew R.M., Hauck J., Olsen A., Peters G.P., Peters W., Pongratz J., Sitch S. (2020). Global Carbon Budget 2020. Earth Syst. Sci. Data.

[9] Cai Z., Yang X., Lin H., Yang X., Jiang P. (2022). Study on the Co-Benefits of Air Pollution Control and Carbon Reduction in the Yellow River Basin: An Assessment Based on a Spatial Econometric Model. Int. J. Environ. Res. Public Health.

[10] Ayres R.U. (1991). The greenhouse effect: Damages, costs and abatement. Environ. Resour. Econ..

[11] IPCC (2001). Climate Change 2001-Mitigation.

[12] Mohan P.S. (2022). Implementing nationally determined contributions under the Paris agreement: An assessment of climate finance in Caribbean small island developing states. Clim. Policy.

[13] Chen L., Wang D., Shi R. (2022). Can China’s Carbon Emissions Trading System Achieve the Synergistic Effect of Carbon Reduction and Pollution Control?. Int. J. Environ. Res. Public Health.

[14] Geroe S. (2019). Addressing Climate Change Through a Low-Cost, High-Impact Carbon Tax. J. Environ. Dev..

[15] Li Q., Shi X., Wu Q. (2021). Effects of China’s ecological restoration on economic development based on Night-Time Light and NDVI data. Environ. Sci. Pollut. Res..

[16] Song J., Wang J., Chen Z. (2022). How Low-Carbon Pilots Affect Chinese Urban Energy Efficiency: An Explanation from Technological Progress. Int. J. Environ. Res. Public Health.

[17] Shigetomi Y., Kanemoto K., Yamamoto Y., Kondo Y. (2021). Quantifying the Carbon Footprint Reduction Potential of Lifestyle Choices in Japan. Environ. Res. Lett..

[18] Sofia D., Gioiella F., Lotrecchiano N., Giuliano A. (2020). Mitigation strategies for reducing air pollution. Environ. Sci. Pollut. Res..

[19] Guillerm N., Cesari G. (2015). Fighting ambient air pollution and its impact on health: From human rights to the right to a clean environment. Int. J. Tuberc. Lung Dis..

[20] Xu B., Lin B. (2016). Regional differences in the CO2 emissions of China’s iron and steel industry: Regional heterogeneity. Energy Policy.

[21] Ramanathan V., Xu Y. (2010). The Copenhagen Accord for limiting global warming: Criteria, constraints, and available avenues. Proc. Natl. Acad. Sci. USA.

[22] Vandyck T., Keramidas K., Kitous A., Spadaro J.V., Van Dingenen R., Holland M., Saveyn B. (2018). Air quality co-benefits for human health and agriculture counterbalance costs to meet Paris Agreement pledges. Nat. Commun..

[23] Anser M.K., Usman M., Godil D.I., Shabbir M.S., Tabash M.I., Ahmad M., Zamir A., Lopez L.B. (2022). Does air pollution affect clean production of sustainable environmental agenda through low carbon energy financing? evidence from ASEAN countries. Energy Environ..

[24] Yan J., Zhao J., Yang X., Su X., Wang H., Ran Q., Shen J. (2021). Does Low-Carbon City Pilot Policy Alleviate Urban Haze Pollution? Empirical Evidence from a Quasi-Natural Experiment in China. Int. J. Environ. Res. Public Health.

[25] Jin S., Wang W., Qalati S.A., Zhang C., Lu N., Zhu G., Wu J. (2022). Can Low-Carbon Technological Innovation Reduce Haze Pollution?—Based on Spatial Econometric Analysis. Front. Environ. Sci..

[26] Okorn K., Jimenez A., Collier-Oxandale A., Johnston J.E., Hannigan M. (2021). Characterizing methane and total non-methane hydrocarbon levels in Los Angeles communities with oil and gas facilities using air quality monitors. Sci. Total Environ..

[27] Peng W., Yang J., Wagner F., Mauzerall D.L. (2017). Substantial air quality and climate co-benefits achievable now with sectoral mitigation strategies in China. Sci. Total Environ..

[28] Wang J., Yu S., Li M., Cheng Y., Wang C. (2022). Study of the Impact of Industrial Restructuring on the Spatial and Temporal Evolution of Carbon Emission Intensity in Chinese Provinces—Analysis of Mediating Effects Based on Technological Innovation. Int. J. Environ. Res. Public Health.

[29] Akhtar M.Z., Zaman K., Rehman F.U., Nassani A.A., Haffar M., Abro M.M.Q. (2022). Evaluating pollution damage function through carbon pricing, renewable energy demand, and cleaner technologies in China: Blue versus green economy. Environ. Sci. Pollut. Res..

[30] Zhou X., Zhang J., Li J. (2013). Industrial structural transformation and carbon dioxide emissions in China. Energy Policy.

[31] Tran Q.T., Huynh N. (2022). Trading-off between being contaminated or stimulated: Are emerging countries doing good jobs in hosting foreign resources?. J. Clean. Prod..

[32] Zou W., Zhang L., Xu J., Xie Y., Chen H. (2022). Spatial–Temporal Evolution Characteristics and Influencing Factors of Industrial Pollution Control Efficiency in China. Sustainability.

[33] Anderson V., Gough W.A. (2021). Nature-Based Resilience: A Multi-Type Evaluation of Productive Green Infrastructure in Agricultural Settings in Ontario, Canada. Atmosphere.

[34] Di Q., Chen X., Hou Z. (2022). Regional differences and key pathway identification of the coordinated governance of pollution control and carbon emission reduction in the three major urban agglomerations of China under the Double-Carbon targets. Resour. Sci..

[35] Guo F., Wang Z., Ji S., Lu Q. (2022). Influential Nodes Identification in the Air Pollution Spatial Correlation Weighted Networks and Collaborative Governance: Taking China’s Three Urban Agglomerations as Examples. Int. J. Environ. Res. Public Health.

[36] Li T., Zheng X., Zhang C., Wang R., Liu J. (2022). Mining Spatial Correlation Patterns of the Urban Functional Areas in Urban Agglomeration: A Case Study of Four Typical Urban Agglomerations in China. Land.

[37] An S., Zhang S., Hou H., Zhang Y., Xu H., Liang J. (2022). Coupling Coordination Analysis of the Ecology and Economy in the Yellow River Basin under the Background of High-Quality Development. Land.

[38] Zheng C., Zhang H., Cai X., Chen L., Liu M., Lin H., Wang X. (2021). Characteristics of CO2 and atmospheric pollutant emissions from China’s cement industry: A life-cycle perspective. J. Clean. Prod..

[39] Chen W., Shen Y., Wang Y. (2018). Evaluation of economic transformation and upgrading of resource-based cities in Shaanxi province based on an improved TOPSIS method. Sustain. Cities Soc..

[40] Fatima T., Xia E., Cao Z., Khan D., Fan J.-L. (2019). Decomposition Analysis of Energy-Related CO2 Emission in the Industrial Sector of China: Evidence from the LMDI Approach. Environ. Sci. Pollut. Res..

[41] Cheng R., Li W. (2019). Evaluating environmental sustainability of an urban industrial plan under the three-line environmental governance policy in China. J. Environ. Manag..

[42] Zhang B., Lu D., He Y., Chiu Y.-H. (2018). The efficiencies of resource-saving and environment: A case study based on Chinese cities. Energy.

[43] Duro J.A., Padilla E. (2011). Inequality across countries in energy intensities: An analysis of the role of energy transformation and final energy consumption. Energy Econ..

[44] Numpacharoen K., Atsawarungruangkit A. (2012). Generating correlation matrices based on the boundaries of their coefficients. PLoS ONE.

[45] Long X., Wu S., Wang J., Wu P., Wang Z. (2022). Urban water environment carrying capacity based on VPOSR-coefficient of variation-grey correlation model: A case of Beijing, China. Ecol. Indic..

[46] Zhang Y.-Y., Zhou H.-T., Younis I., Zhou L. (2021). Coupling Coordination Analysis of Technological Innovation, Standards, and Quality: Evidence From China. SAGE Open.

[47] Guo Y., Hu F., Xie J., Liu C., Yang Y., Ding H., Wu X. (2022). Data-Driven Evaluation of the Synergetic Development of Regional Carbon Emissions in the Yangtze River Delta. Processes.

[48] Bai L., Chen H., Gao Q., Luo W. (2018). Project portfolio selection based on synergy degree of composite system. Soft Comput..

[49] Chen J., Xu C., Cui L., Huang S., Song M. (2019). Driving factors of CO2 emissions and inequality characteristics in China: A combined decomposition approach. Energy Econ..

[50] Luan B., Zou H., Chen S., Huang J. (2021). The effect of industrial structure adjustment on China’s energy intensity: Evidence from linear and nonlinear analysis. Energy.

[51] Yu Y., Liu H. (2020). Economic growth, industrial structure and nitrogen oxide emissions reduction and prediction in China. Atmos. Pollut. Res..

[52] Sadorsky P. (2013). Do urbanization and industrialization affect energy intensity in developing countries?. Energy Econ..

[53] Collaborator P., Chatterjee C. (2017). Does environmental regulation indirectly induce upstream innovation?. New Evid. India. Res. Policy.

[54] Mulaessa N., Lin L. (2021). How do proactive environmental strategies affect green innovation? The moderating role of environmental regulations and firm performance. Int. J. Environ. Res. Public Health.

[55] Chen H., Hao Y., Li J., Song X. (2018). The impact of environmental regulation, shadow economy, and corruption on environmental quality: Theory and empirical evidence from China. J. Clean. Prod..

[56] Namahoro J.P., Wu Q., Xiao H., Zhou N. (2021). The Impact of Renewable Energy, Economic and Population Growth on CO_2_ Emissions in the East African Region: Evidence from Common Correlated Effect Means Group and Asymmetric Analysis. Energies.

[57] Wang Y., Zou H., Duan X., Wang L. (2022). Coordinated Evolution and Influencing Factors of Population and Economy in the Yangtze River Economic Belt. Int. J. Environ. Res. Public Health.

[58] Mahmood T., Ahmad E. (2018). The relationship of energy intensity with economic growth: Evidence for European economies. Energy Strategy Rev..

[59] Wang X., Zhang Q., Chang W.Y. (2022). Does economic agglomeration affect haze pollution? Evidence from China’s Yellow River basin. J. Clean. Prod..

[60] Emir F., Bekun F.V. (2019). Energy intensity, carbon emissions, renewable energy, and economic growth nexus: New insights from Romania. Energy Environ..

[61] Hu W., Fan Y. (2020). City size and energy conservation: Do large cities in China consume more energy?. Energy Econ..

[62] Otsuka A., Goto M., Sueyoshi T. (2014). Energy efficiency and agglomeration economies: The case of Japanese manufacturing industries. Reg. Sci. Policy Pract..

[63] Bilgili F., Koçak E., Bulut Ü., Kulo glu A. (2017). The impact of urbanization on energy intensity: Panel data evidence considering cross-sectional dependence and heterogeneity. Energy.

[64] Razmjoo A., Gakenia Kaigutha L., Vaziri Rad M.A., Marzband M., Davarpanah A., Denai M. (2021). A Technical analysis investigating energy sustainability utilizing reliable renewable energy sources to reduce CO_2_ emissions in a high potential area. Renew. Energy.

[65] Simbi C.H., Lin J., Yang D., Ndayishimiye J.C., Liu Y., Li H., Xu L., Ma W. (2021). Decomposition and Decoupling Analysis of Carbon Dioxide Emissions in African Countries during 1984–2014. J. Environ. Sci..

[66] Li X.M., Zhou W.Q. (2019). Optimizing urban greenspace spatial pattern to mitigate urban heat island effects: Extending understanding from local to the city scale. Urban For. Urban Gree..

[67] Yu Z.W., Yang G.Y., Zuo S.D., Jørgensen G., Koga M., Vejre H. (2020). Critical review on the cooling effect of urban blue-green space: A threshold-size perspective. Urban For. Urban Green..

[68] Cole M.A., Elliott R.J.R., Okubo T. (2010). Trade, environmental regulations and industrial mobility: An industry-level study of Japan. Ecol. Econ..

[69] Liobikienė G., Butkus M. (2019). Scale, composition, and technique effects through which the economic growth, foreign direct investment, urbanization, and trade affect greenhouse gas emissions. Renew. Energy.

